# Use of Chronic Prescription Medications and Prevalence of Polypharmacy in Survivors of Childhood Cancer

**DOI:** 10.3389/fonc.2021.642544

**Published:** 2021-04-01

**Authors:** Celeste L. Y. Ewig, Yi Man Cheng, Hoi Shan Li, Jasper Chak Ling Wong, Alex Hong Yu Cho, Freddie Man Hong Poon, Chi Kong Li, Yin Ting Cheung

**Affiliations:** ^1^School of Pharmacy, Faculty of Medicine, The Chinese University of Hong Kong, Hong Kong, China; ^2^Department of Pharmacy, Hong Kong Children’s Hospital, Hong Kong, China; ^3^Department of Paediatrics, Faculty of Medicine, The Chinese University of Hong Kong, Hong Kong, China; ^4^Hong Kong Hub of Paediatric Excellence, The Chinese University of Hong Kong, Hong Kong, China; ^5^Department of Oncology and Hematology, The Hong Kong Children’s Hospital, Hong Kong, China

**Keywords:** childhood cancer survivor, pediatric oncology, chronic medication prescriptions, polypharmacy, medication utilization

## Abstract

**Background:**

As survivors of childhood cancer age, development of cancer treatment-related chronic health conditions often occur. This study aimed to describe the pattern of chronic prescription medication use and identify factors associated with polypharmacy among survivors of childhood cancer.

**Methods:**

This was a retrospective study conducted at the pediatric oncology long-term follow-up clinic in Hong Kong. Eligible subjects included survivors who were (1) diagnosed with cancer before 18 years old, (2) were at least 3 years post-cancer diagnosis and had completed treatment for at least 30 days, and (3) receiving long-term follow-up care at the study site between 2015 and 2018. Dispensing records of eligible survivors were reviewed to identify medications taken daily for ≥30 days or used on an “as needed” basis for ≥6 months cumulatively within the past 12-month period. Polypharmacy was defined as the concurrent use of ≥5 chronic medications. Multivariable log-binomial modeling was conducted to identify treatment and clinical factors associated with medication use pattern and polypharmacy.

**Results:**

This study included 625 survivors (mean current age = 17.9 years, standard deviation [SD] = 7.2 years) who were 9.2 [5.2] years post-treatment. Approximately one-third (n = 219, 35.0%) of survivors were prescribed at least one chronic medication. Frequently prescribed medication classes include systemic antihistamines (26.5%), sex hormones (19.2%), and thyroid replacement therapy (16.0%). Overall prevalence of polypharmacy was 5.3% (n = 33). A higher rate of polypharmacy was found in survivors of CNS tumors (13.6%) than in survivors of hematological malignancies (4.3%) and other solid tumors (5.3%) (*P* = .0051). Higher medication burden was also observed in survivors who had undergone cranial radiation (RR = 6.31; 95% CI = 2.75–14.49) or hematopoietic stem-cell transplantation (HSCT) (RR = 3.53; 95% CI = 1.59–7.83).

**Conclusion:**

Although polypharmacy was observed in a minority of included survivors of childhood cancer, chronic medication use was common. Special attention should be paid to survivors of CNS tumors and survivors who have undergone HSCT or cranial radiation. These individuals should be monitored closely for drug–drug interactions and adverse health outcomes that may result from multiple chronic medications, particularly during hospitalization in an acute care setting.

## Introduction

Advances in medical knowledge and treatment of cancer have improved the 5-year survival rate of patients with childhood cancer from 50% three decades ago to more than 80% at present (1, 2). However, cancer survivorship comes with a cost of developing a myriad of chronic health conditions as the late adverse effects of cancer treatment (3–7). It is reported that more than three-quarters of childhood cancer survivors suffer from at least one late effect within 35 years after the end of treatment (4). Survivors of childhood cancer are also eight times more likely to develop a severe or life-threatening chronic condition such as cardiomyopathy, premature gonadal failure, metabolic syndrome, and neurocognitive dysfunction (3).

Management of these late effects may require lifelong medications. Among adult cancer survivors, the reported prevalence of polypharmacy, i.e., the concurrent use of multiple chronic medications, ranges from 44.4% to 64.0%, which is considerably higher than that in age-matched non-cancer controls and the general population (8, 9). However, limited epidemiological studies have been conducted to evaluate medication use patterns among survivors of childhood cancer, who may be even more susceptible to the premature onset of late effects of cancer treatment and non-cancer-related medical conditions. One study reported that central nervous system (CNS) agents, hormone replacement therapy, and anti-infectives were more frequently prescribed to survivors of childhood cancer than to non-cancer controls (10). Similarly, compared with their non-cancer siblings, adolescent and adult survivors of childhood cancer were found to have a higher utilization of psychoactive prescription medications for managing pain and psychological symptoms (11, 12).

Unfortunately, although pharmacological intervention may confer therapeutic benefits to patients with multiple chronic conditions, an increased use of chronic medications increases the odds of having polypharmacy. It is well established in the literature that polypharmacy is associated with unwanted consequences such as an increased risk of drug–drug interactions, adverse drug events, and medication non-adherence in cancer patients (8, 13, 14). Mitigating these negative outcomes requires identifying subgroups of patients who are at risk of polypharmacy so their medication regimen can be improved. Certain late effects can occur early within a few years after completing cancer treatment (15), raising the question of whether the use of chronic medications is more prevalent among adolescent and young adult survivors of cancer than in the general population. Furthermore, little is reported about the prevalence and predictors of polypharmacy in survivors of childhood cancer.

The primary objectives of this study were to describe the pattern of chronic prescription medication use and estimate the prevalence of polypharmacy in survivors of childhood cancer. The secondary objective was to identify the clinical and treatment factors associated with medication burden and polypharmacy.

## Methods

### Study Design and Setting

This was a retrospective cross-sectional study conducted between January 2019 and December 2019 at the Long-term Follow-up (LTFU) Clinic of the Prince of Wales Hospital in Hong Kong, a regional tertiary care public hospital that serves as one of the major hubs for providing LTFU care for survivors of childhood cancer.

Data on the treatment history and prescription medication use of the eligible survivors were extracted from the internal data repository and electronic patient record system of the study institution, known as the Clinical Data Analysis and Reporting System (CDARS) and Clinical Management System (CMS), respectively, and were retrospectively reviewed. The CDARS database includes patient-specific data, such as the clinical diagnosis, prescribed and dispensed medications, and information on hospitalization and discharge, that are entered by trained clinicians and healthcare professionals. Both the CDARS and CMS databases have been proven to be reliable sources of health administrative data for conducting epidemiological research in Hong Kong (16, 17). This study was approved by the Joint Chinese University of Hong Kong – New Territories East Cluster Clinical Research Ethics Committee (Ref. No. 2018.427).

### Study Population and Setting

Survivors were eligible if they were diagnosed with cancer before the age of 18 years between January 1, 2000, and December 31, 2015. We included survivors who were diagnosed with cancer before the age of 18 years, as patients of this age range are typically treated under pediatric specialty care in the Hong Kong healthcare system. Patients who were diagnosed prior to January 2000 were also excluded as the electronic CMS was in full operation only after 2000.

Eligible survivors were also (1) at least 3 years post-cancer diagnosis; (2) considered “end-of-treatment (EOT),” which is defined as more than 30 days from the last documented date of chemotherapy, immunotherapy, targeted therapy, radiation, or surgery; and (3) receiving active LTFU care at the study site between December 1, 2015, and September 30, 2018. This definition of survivorship was adopted to examine drug utilization in survivors from the early post-therapy phase to the long-term survivorship phase. This definition was also adopted in a similar study [Smitherman et al. (10)]. We excluded patients who (1) did not receive cancer treatment at the study institution; (2) developed secondary malignancies; or (3) were deceased at the time of data collection.

### Study Outcomes

The primary outcomes were medication burden (the number of chronic prescription medications) and polypharmacy. Chronic medications were defined as medications prescribed to be taken daily for ≥30 days or used on an “as needed” basis for ≥6 months cumulatively within the past 12-month period. Each dispensed medication was counted as one medication item, regardless of the number of active ingredients. Medications were categorized according to the Anatomical Therapeutic Chemical Classification from WHO Collaborating Centre for Drug Statistics Methodology (18). Medications without active ingredients were excluded (**Supplement 1**).

For this study, we adopted the most popular and well-accepted definition of polypharmacy for the overall cohort, i.e., the use of ≥5 concurrent chronic medications (19).

### Predictors

Based on a literature review (9–11), several predictive factors for chronic medication use were identified *a priori*. Clinical predictors included age at diagnosis, primary cancer diagnosis, chronic conditions, and years since EOT. Treatment predictors included chemotherapy, radiotherapy, surgery, and hematopoietic stem-cell transplantation (HSCT).

### Data Analysis

Descriptive statistics were used to summarize the distribution of relevant outcome variables, predictors, and covariates according to reasonable groupings that are consistent with those in previous reports on childhood cancer (10, 11). Multivariable log-binomial models (generalized linear models with Poisson error and log-link function) were used to identify clinical and treatment factors associated with polypharmacy adjusted for sex and current age. Relative risk (RR) estimates and 95% confidence intervals (CIs) were reported. A similar multivariable log-binomial model was also used to identify clinical and treatment factors associated with specific therapeutic classes of chronic medications. Only the therapeutic classes used by more than 10% of the cohort were included in the analyses.

Finally, a Cox proportional hazards regression analysis was conducted to calculate hazard ratios (HRs) with 95% CIs for polypharmacy across years since diagnosis. HRs are presented for the variables identified in the previous multivariable log-binomial models for predicting polypharmacy, adjusting for sex. The Schoenfeld residuals were calculated for each variable to ensure that it independently satisfied the assumptions of the Cox model.

A series of sensitivity analyses were conducted. (1) Emerging evidence suggests an alternative definition of polypharmacy in the pediatric population, i.e., the use of ≥2 concurrent chronic medications (20). For a sensitivity analysis, we defined polypharmacy by this alternative definition in survivors aged less than 18 years. (2) Different international childhood cancer cohorts adopt varying diagnosis ages for childhood cancer, which include 0 to 15 years [British Childhood Cancer Survivor Study (21)], 0 to 18 years [Late Effects of Childhood Cancer task force of the Dutch Childhood Oncology Group (22) and Canadian PETALE (23)], and 0 to 21 years [United States Childhood Cancer Survivor Study (24) and Swiss Childhood Cancer Survivor Study (25)]. For a sensitivity analysis, we reported the rate of polypharmacy for survivors who were diagnosed at 0 to 15 years (“pediatric” cancer) and >15 years to 18 years (“adolescent” cancer). (3) The rate of polypharmacy was also reported separately for “early survivors” vs. “long-term” survivors (defined as ≤ 5 years post-diagnosis and > 5 years post-diagnosis, respectively). Association between age at diagnosis and time since diagnosis, adopting the above definitions in (2) and (3), was evaluated using a similar multivariable log-binomial model for sensitivity analysis. A *P* value of less than.05 was considered statistically significant, and all tests were two-sided. All analyses were conducted in SAS (SAS 9.4, SAS Institute, Cary, NC).

## Results

### Study Population Characteristics

**Figure 1:**
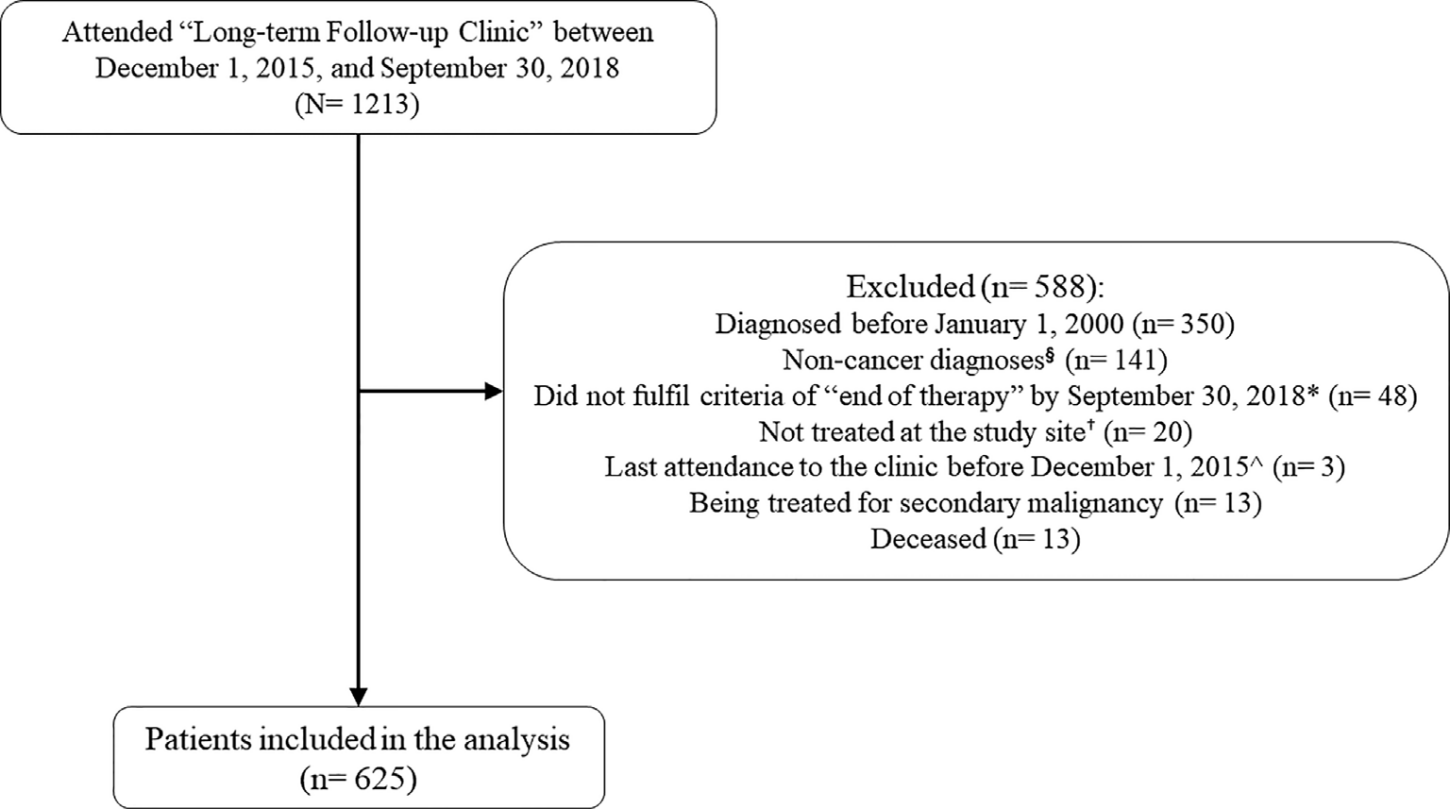
Subject screening. ^§^ The “Long-term Follow-Up Clinic” of the study site (Prince of Wales Hospital) provides follow-up care for survivors of pediatric cancers and hematology diseases. Patients who were diagnosed with non-cancer conditions (e.g. aplastic anemia, thalassemia, etc.) were excluded. *“End-of-treatment” is defined as more than 30 days from the last documented date of chemotherapy, immunotherapy, targeted therapy, radiation, or surgery + Survivors not treated at the study site was excluded as we were not able to retrieve their treatment history. ^Survivors with stable health status are typically scheduled for a follow-up consultation once every 1 to 2.5 years. Survivors whose last attendance to the clinic was before December 2015 were likely lost to follow up.

A total of 1213 patients were screened for eligibility (**Figure 1**). The final study population included 625 survivors of childhood cancer (mean current age = 17.9 years, standard deviation [SD] = 7.2 years) whose time since EOT was 9.2 [5.2] years (**Table 1**). The mean age at cancer diagnosis was 7.6 (5.34) years. The single largest group of survivors was diagnosed with leukemia (n = 275, 44.0%), followed by lymphoma (n = 70, 11.2%) and central nervous system (CNS) tumor (n = 66, 10.6%). The major treatment modalities were chemotherapy (n = 557, 89.1%) and surgery (n = 265, 42.4%). Among survivors who had undergone radiation therapy (n = 148, 23.7%), almost half had received cranial radiation (n = 73, 49.3%), followed by chest (n = 21, 14.2%) and abdominal (n = 19, 12.8%) radiation. Approximately one-tenth of the survivors (n = 68, 10.9%) suffered from at least one chronic health condition at the time of evaluation.

**Table 1:** Characteristics of study population (n = 625).

Characteristics	N (%)
**Sex**	
Male	358 (57.3)
Female	267 (42.7)
**Current Age, years**	
Mean (± SD)	17.9 (± 7.24)
≥3 to ≤ 6	22 (3.5)
>6 to ≤ 12	134 (21.4)
>12 to ≤ 18	146 (23.4)
>18 to ≤ 30	306 (48.9)
>30	17 (4.7)
**Age at Diagnosis, years**	
Mean (± SD)	7.6 (± 5.35)
Pediatric cancer:*	
0 to ≤ 6	300 (48.0)
>6 to ≤ 12	155 (24.8)
>12 to < 15	97 (15.5)
Adolescent cancer:*	
≥ 15 to ≤ 18	73 (11.7)
**Time since Cancer Diagnosis, years**	
Mean (± SD)	10.4 (± 5.2)
≤5 years post-diagnosis:*	
≤ 3	37 (5.9)
> 3 to 5	105 (16.8)
>5 years post-diagnosis:*	
>5 to 10	174 (27.8)
>10 to 15	148 (23.7)
>15	161 (25.8)
**Time since End of Treatment, years**	
Mean (± SD)	9.2 (± 5.18)
≤2	55 (8.8)
≤2 to 5	115 (18.4)
>5 to ≤ 10	175 (28.0)
>10 to ≤ 15	159 (25.4)
>15	121 (19.4)
**Primary Cancer Diagnosis**	
**Hematological**	
Leukemia	275 (44.0)
Lymphoma	70 (11.2)
**CNS tumors**	66 (10.6)
**Other solid tumors**	
Malignant bone tumors	48 (7.7)
Germ cell tumors	40 (6.4)
Neuroblastoma and other peripheral nervous cell tumors	33 (5.3)
Soft tissue carcinoma	32 (5.1)
Renal carcinoma	25 (4.0)
Other malignant epithelial neoplasms and malignant melanomas	14 (2.2)
Hepatic tumors	13 (2.1)
Retinoblastoma	5 (0.8)
Other and unspecified malignant neoplasms	4 (0.6)
**Treatment**	
**Chemotherapy**	557 (89.1)
Alkylating agents	483 (86.7)
Mustard gas derivatives	402 (72.2)
Heavy metals	158 (28.4)
Antitumor Antibiotics	476 (85.5)
Anthracyclines	430 (77.2)
Plant Alkaloids	380 (68.2)
Vinca Alkaloids	378 (67.9)
Antimetabolites	376 (67.5)
Folic acid antagonist	342 (61.4)
Pyrimidine antagonist	325 (58.4)
Purine antagonist	262 (47.0)
Topoisomerase inhibitors	238 (42.7)
**Surgery**	265 (42.4)
**Radiotherapy**	148 (23.7)
Cranial	73 (49.3)
Other body sites	75 (12.0)
Chest	21 (14.2)
Abdominal	19 (12.8)
Pelvic	16 (10.8)
Others	21 (3.4)
Total body	19 (12.8)
**Bone marrow transplant**	75 (12.0)
**Other cancer therapies**^§^	43 (7.7)
**Chronic health conditions**	68 (10.9)
Endocrine^	62 (9.9)
Metabolism	9 (1.4)
Neurological	5 (0.8)
Psychiatry	

*Subgroups and definitions adopted for sensitivity analyses.

^§^Includes immunotherapy, targeted therapy, hormonal therapy and other types of treatment.

^Includes hypopituitarism, growth hormone deficiency, gonadal dysfunction, thyroid dysfunction.

### Pattern of Chronic Medication Use and Polypharmacy

Approximately one-third of the survivors (n = 219, 35%) were prescribed at least one chronic medication (**Table 2**). Among these survivors, the median number of medications was 2 (interquartile range: 1–3). The most commonly prescribed medications belonged to the therapeutic classes of systemic antihistamines (n = 58, 26.5%), sex hormone replacement therapy (n = 42, 19.2%), thyroid therapy (n = 35, 16.0%), and systemic antimicrobials (n = 33, 15.1%). **Supplement 2** summarizes the therapeutic classes and specific medications prescribed to more than 5% of the cohort prescribed at least one chronic medication.

**Table 2:** Pattern of prescription chronic medication use.

Overall cohort	N = 625
**Use of prescription chronic medication**	**n (%)**
Patients with ≥1 chronic medication	219 (35.0)
Patients with no chronic medication	406 (65.0)
**Subgroup with prescribed chronic medication**	n = 219
**No. of concurrent chronic medications per patient**	
Median (interquartile range) [range]	2 (1–3) [1–22]
1 to 2	143 (65.3)
3 to 4	43 (19.6)
5 to 6	20 (9.1)
7 to 8	7 (3.2)
>9	6 (2.7)
Therapeutic classification^*^	
Systemic antihistamines	58 (26.5)
Sex hormones replacement therapy	42 (19.2)
Thyroid therapy	35 (16.0)
Systemic antimicrobials	33 (15.1)
Drugs for obstructive airway diseases	21 (9.6)
Drugs for acid-related gastric disorders	17 (7.8)
Pituitary and hypothalamic hormones and analog	16 (7.3)

*Includes the therapeutic classes prescribed to more than 5% of the cohort prescribed with chronic medications (n = 219).

**Figure 2:**
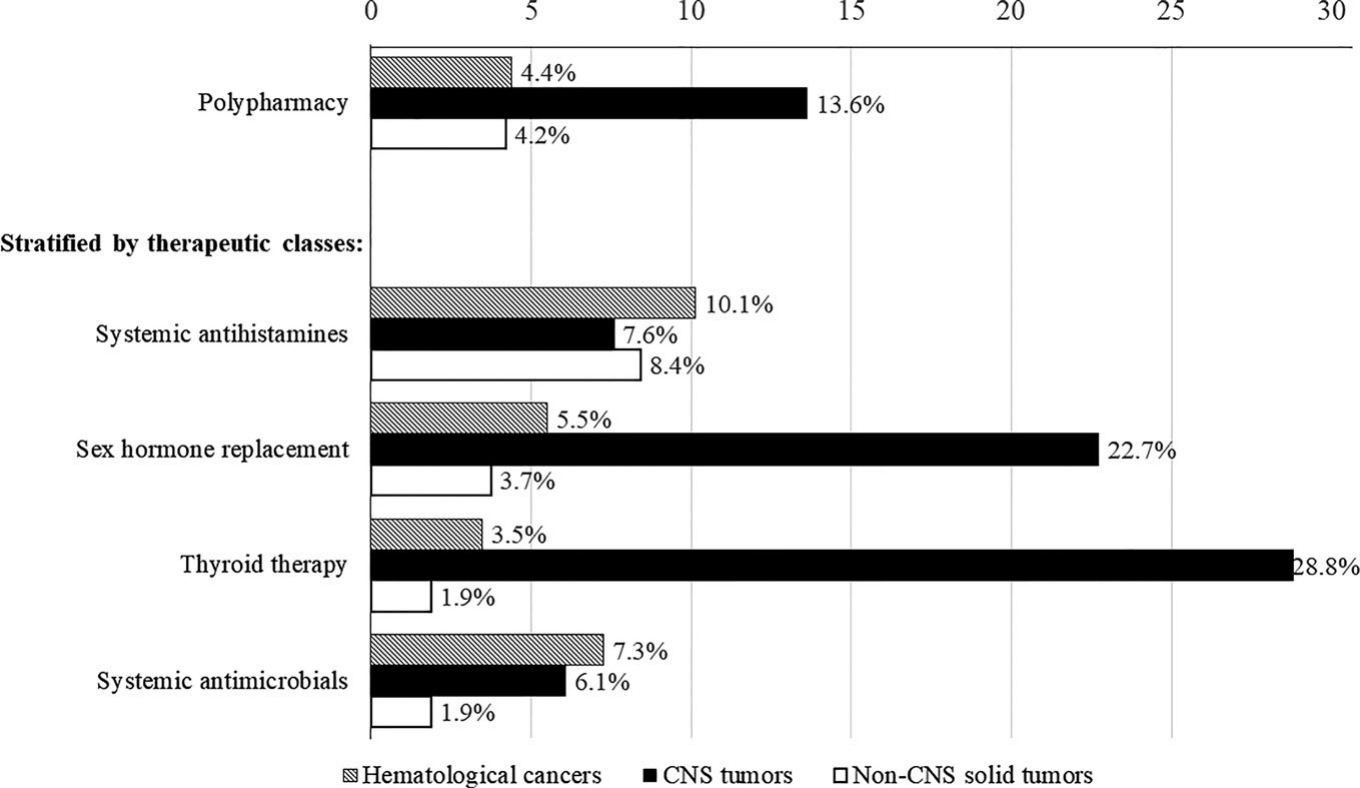
Rates of polypharmacy and medication use stratified by cancer diagnoses. Includes only therapeutic classes used by more than 10% of survivors prescribed with chronic medications.

Collectively, the prevalence of polypharmacy (≥5 concurrent chronic medications) was 5.3% (n = 33) in the overall study cohort and 15.1% among survivors with chronic medication use. The rates of polypharmacy in clinically relevant subgroups of survivors are presented in **Supplement 3**. Polypharmacy was observed in 4.5%–5.9% of survivors aged less than 18 years, 4.9% of survivors aged 19–30 years, and 11.7% of survivors aged more than 30 years. In terms of cancer diagnosis, the rates of polypharmacy were 4.4%, 4.2%, and 13.6% in survivors of hematological malignancies, solid tumors, and CNS tumors, respectively (**Figure 2**). The rates of polypharmacy for “early survivors” (≤ 5 years post-diagnosis) and long-term survivors (>5 years post-diagnosis) were 7.0% and 4.8%, respectively (**Supplement 3**). Polypharmacy was also observed in a minority of survivors who had received radiation to the CNS (16.4%) and other body sites (5.3%), as well as in survivors who had undergone HSCT (13.3%).

When we adopted the alternative definition of polypharmacy (≥2 concurrent chronic medications) in the pediatric population, the proportion of pediatric survivors taking chronic medications experiencing polypharmacy and the rate of polypharmacy in the overall study cohort were found to be 22.5% and 15.9% (n = 99), respectively.

### Factors Associated With Polypharmacy

Survivors of CNS tumors were more likely to have polypharmacy than survivors of non-CNS solid tumors (RR = 4.10, 95% CI = 1.52–11.0) (**Table 3**). Previous exposures to cranial radiation (RR = 6.31, 95% CI = 2.75–14.49) and HSCT (RR = 3.53, 95% CI = 1.59–7.83) were also associated with higher odds of having polypharmacy. Survivors who developed chronic health conditions had 7 times higher odds of having polypharmacy than survivors who did not have chronic conditions (RR = 7.35, 95% CI = 3.45–15.66).

**Table 3:** Factors associated with polypharmacy.

	Risk Ratio	95% CI		*P*
**Demographic factors**				
**Sex**				
Male	0.60	0.30	1.22	0.16
Female	*Ref*			
**Current age at study**	1.01	0.96	1.01	0.69
Clinical factors*				
**Age at diagnosis**	1.05	0.96	1.15	0.22
**Years since EOT**	0.93	0.85	1.02	0.15
**Primary diagnosis**				
Hematological	1.04	0.44	2.45	0.91
CNS	4.10	1.52	11.02	**0.0051**
Non-CNS solid tumor	*Ref*			
**Chronic health conditions (any)**				
Yes	7.35	3.45	15.66	**<.0001**
No	*Ref*			
Treatment factors*				
**Chemotherapy**				
Yes	0.65	0.24	1.77	0.40
No	*Ref*			
**Radiation**				
Cranial radiation	6.31	2.75	14.49	**<.0001**
Body only (Chest, abdomen, pelvis)	1.60	0.51	4.91	0.41
None	*Ref*			
**Surgery**				
Yes	1.13	0.56	2.30	0.72
No	*Ref*			
**Bone Marrow Transplant**				
Yes	3.53	1.59	7.83	**0.0019**
No	*Ref*			

CNS, central nervous system; EOT, end of treatment; Ref, reference group.

*Models are adjusted for demographic factors: sex and age at study. Bold: Statistical significance P < 0.05.

For the multivariable Cox model (**Supplement 4**), the adjusted hazard ratios (HRs) for polypharmacy across years since cancer diagnosis showed significant association between polypharmacy and CNS tumor diagnosis (HR = 1.79, 95% CI = 1.25–5.07), chronic health conditions (HR = 3.39, 95% CI = 1.57–7.34), cranial radiation (HR = 3.34, 95% CI = 1.22 – 9.13) and bone marrow transplant (HR = 2.58, 95% CI = 1.19–5.62).

### Factors Associated With Specific Therapeutic Classes of Medications

Subsequent analyses were performed to identify factors associated with the use of specific therapeutic classes of medications prescribed to more than 10% of the cohort (**Table 3**). The therapeutic classes included systemic antihistamines, sex hormone replacement therapy, thyroid therapy, and systemic antimicrobials. Older age was associated with the use of sex hormone replacement therapy (RR = 1.11, 95% CI = 1.06–1.17) and thyroid therapy (RR = 1.07, 95% CI = 1.01–1.12), whereas younger age was associated with the use of systemic antihistamines (RR = 0.94, 95% CI = 0.91–0.98) and antimicrobials (RR = 0.87, 95% CI = 0.82–0.92).

Compared with survivors of non-CNS solid tumors, survivors of CNS tumors were more likely to be prescribed sex hormone replacement therapy (RR = 9.98, 95% CI = 3.74–26.66) and thyroid therapy (RR = 23.83, 95% CI = 7.45–76.20) (**Table 4**). More than one-fifth of CNS tumor survivors were prescribed sex hormone (22.7%) and thyroid (28.8%) therapies, as compared with less than 5% of survivors of other cancers (**Figure 2**). In contrast, survivors of hematological malignancies were more likely than survivors of solid tumors to be prescribed systemic antimicrobials (RR = 6.72, 95% CI = 2.20–20.53) (**Table 4**). Having at least one chronic condition was associated with higher odds of receiving sex hormone replacement therapy (RR = 24.43, 95% CI = 11.6–51.45) and thyroid therapy (RR = 36.96, 95% CI = 16.06–85.02).

**Table 4:** Factors associated with specific therapeutic classes of medications.

	Systemic antihistamines				Sex hormone replacement				Thyroid therapy				Systemic antimicrobials			
	RR	95% CI		*P*	RR	95% CI		*P*	RR	95% CI		*P*	RR	95% CI		*P*
**Demographic factors**																
**Sex**																
Male	0.96	0.55	1.66	0.88	0.81	0.43	1.54	0.53	1.30	0.64	2.64	0.46	1.23	0.58	2.60	0.57
Female	*Ref*				*Ref*											
**Current age at study**	0.94	0.91	0.98	**0.0061**	1.11	1.06	1.17	**<.0001**	1.07	1.01	1.12	**0.0072**	0.87	0.82	0.92	**<.0001**
Clinical factors*																
**Age at diagnosis**	1.05	0.98	1.13	0.14	0.94	0.87	1.03	0.21	1.02	0.93	1.11	0.64	1.50	1.28	1.76	**<.0001**
**Years since EOT**	0.92	0.86	1.00	0.053	1.04	0.96	1.13	0.31	0.97	0.89	1.06	0.62	0.50	0.39	0.64	**<.0001**
**Primary diagnosis**																
Hematological	1.38	0.75	2.55	0.29	1.44	0.61	3.43	0.39	1.77	0.56	5.61	0.32	6.72	2.20	20.53	**0.0008**
CNS	0.94	0.33	2.68	0.91	9.98	3.74	26.66	**<.0001**	23.83	7.45	76.20	**<.0001**	4.50	1.04	19.34	**0.043**
Non-CNS solid tumor	*Ref*				*Ref*				*Ref*				*Ref*			
**Chronic health conditions (any)**																
Yes	1.54	0.68	3.44	0.29	24.43	11.60	51.45	**<.0001**	36.96	16.06	85.02	**<.0001**	0.72	0.16	3.18	0.66
No	*Ref*				*Ref*				*Ref*				*Ref*			
Treatment factors*																
**Chemotherapy**																
Yes	2.11	0.72	6.12	0.16	0.90	0.30	2.71	0.85	3.56	0.47	26.72	0.21	§			
No					*Ref*				*Ref*				*Ref*			
**Radiation**																
Cranial radiation	0.67	0.25	1.75	0.41	14.15	6.20	32.29	**<.0001**	13.81	5.73	33.27	**<.0001**	1.23	0.40	3.77	0.70
Body only (Chest, abdomen, pelvis)	0.61	0.21	1.80	0.37	6.96	2.85	17.01	**<.0001**	6.75	2.57	17.70	**0.0001**	1.28	0.35	4.59	0.69
None	*Ref*				*Ref*				*Ref*				*Ref*			
**Surgery**																
Yes	0.87	0.50	1.52	0.61	1.21	0.63	2.30	0.56	1.60	0.80	3.19	0.18	2.47	1.11	5.49	**0.026**
No	*Ref*				*Ref*				*Ref*				*Ref*			
**Bone Marrow Transplant**																
Yes	1.15	0.50	2.65	0.75	10.22	5.12	20.38	**<.0001**	3.93	1.84	8.36	**0.0004**	3.97	1.61	9.80	**0.0027**
No	*Ref*				*Ref*				*Ref*				*Ref*			

RR, relative risk; 95% CI, 95% confidence interval; Ref, Reference group.

*Models are adjusted for demographic factors: sex and age at study.

^§^Association analysis was not conducted for variable “chemotherapy” as the sample size for non-chemotherapy receiving survivors treated with systemic antimicrobials was too low, hence yielding unstable parameter estimates. Bold: Statistical significance P < 0.05.

### Sensitivity Analyses

A sensitivity analysis showed that when adopting the alternative definition of polypharmacy (≥2 concurrent chronic medications) in pediatric survivors, the predictors of polypharmacy were similar to those identified in the primary analysis (**Supplement 5**). However, associations became weaker, as reflected by the decrease in the magnitude of the risk ratio estimates (**Supplement 5**). Additional sensitivity analysis also did not show significant association between age at diagnosis (pediatric vs. adolescent) and time since diagnosis (≤ 5 years post-diagnosis *vs.* > 5 years post-diagnosis) with polypharmacy (**Supplement 6**).

## Discussion

Currently, limited drug utilization data on childhood cancer survivors are available in the literature. This study examined the prevalence and predictors of polypharmacy in a cohort of relatively young survivors of childhood cancer. We found that 35% of these young survivors were prescribed at least one chronic medication with polypharmacy detected in a minority of survivors. Our findings are consistent with the well-established evidence that certain subgroups of survivors are at a higher risk of developing multiple cancer treatment-related chronic health conditions that require pharmacological interventions (3, 7, 15, 26). Higher medication burden was found in HSCT recipients, who are known to suffer from a myriad of late effects due to intensive myeloablative conditioning treatment and total body irradiation. Sex hormones and thyroid medications were commonly prescribed to treat secondary endocrinopathy in survivors of CNS tumors and survivors who had undergone cranial radiation. These procedures typically involve higher radiotherapy doses of >30Gy and have a known association with endocrinopathy. Importantly, older age was associated with an increased use of hormone medications, which indicates that cancer survivors develop treatment-related chronic medical morbidities as they age. Survivors of hematological malignancies and CNS tumors were more likely to be persistently prescribed antimicrobials. This may reflect the subset of post-BMT patients with chronic graft-versus-host-disease (GVHD) and a small subgroup of patients who underwent splenectomy.

Our findings are comparable to those of a similar study by Smitherman et al. (10), who evaluated prescription drug use in survivors of childhood cancer in the United States using a commercial insurance claims database. This similarity most likely stems from the comparable contemporary cancer treatments adopted by the United States and Hong Kong. Both studies identified higher utilization of gonadal, pituitary, and thyroid replacement hormones and antimicrobials (antibacterials, antifungals, and antivirals) in survivors. However, it is worth noting that minor differences exist in drug use patterns between the two studies. First, antihistamines were the most commonly prescribed medications in our cohort but not in Smitherman et al.’s study cohort. The high utilization of prescribed systemic antihistamines in younger survivors in our study may be due to several reasons. First, the prevalence of allergies in the local pediatric population is often higher than western countries due to environmental factors. Secondly, systemic antihistamines are often prescribed to local patients during their routine follow-up visits for improved affordability and convenience. In most western countries, these medications are often available as over-the-counter and do not require a prescription for purchase. Second, contrary to Smitherman et al.’s study, we did not identify a high rate of antihypertensive use in our cohort. Cardiovascular morbidities are more prevalent in survivors of lymphoma who are treated with high-dose anthracyclines and thoracic radiation (4, 27, 28). The higher proportion of lymphoma survivors in Smitherman et al.’s study cohort (26.4%) than in our study cohort (11.2%) may explain this difference. Third, Smitherman et al.’s study and reports from the Childhood Cancer Survivors Study (11, 12) in the United States found that survivors were more likely to use opioids and psychoactive medications, which were not common classes of prescription drugs found in our study cohort. We speculate that this difference in drug use pattern may be attributable to the lower utilization of psychotropic drugs and opioids in Asian populations (29, 30). Findings from our study may reflect the late chronic health problems experienced by subgroups of cancer survivors treated with contemporary regimens, and should be validated through a multi-centered study in Hong Kong, as well as collaboration with other international groups (31, 32). For example, the linkage of community-dispensed prescriptions data to cancer registration data in the United Kingdom can greatly enhance our understanding of the patient pathway and drug utilization of cancer patients (33, 34). As cancer treatment strategies evolve, up-to-date assessments of morbidities and medication use patterns are necessary to provide optimal risk-based survivorship care for survivors of childhood cancer.

The rate of polypharmacy (≥5 concurrent chronic medications) in our cohort of young survivors was low. However, it is worth noting that this estimate is higher than the reported rates of polypharmacy in the general population aged less than 40 years. When applying the same definition of polypharmacy, the rate of polypharmacy in individuals aged 20–39 years was 0.3% in one Dutch study (35) and 3.1% in one study from the United States (36). An Italian study reported that polypharmacy was detected in 2.98% and 3.29% of female and male subjects aged 15–64 years, respectively (37). In the general population of Japan, the rate of polypharmacy was 4.9% in those aged 20–34 years (38). All of these reported estimates from the literature seem to be lower than the polypharmacy rate of 5.3% observed in our study cohort. Importantly, we found that the rate of polypharmacy in survivors aged more than 30 years was considerably higher (11.7%). We acknowledge that this finding must be interpreted with caution due to the absence of an appropriate control group. However, it is reasonable to speculate that given the higher reported prevalence of chronic health conditions in childhood cancer survivors of the North American, European and Asian populations (5–7, 15, 22, 39), their medication burden may be considerably higher than that of the general population. An additional point to note is that these are relatively young survivors and they are at risk of developing more chronic morbidities with longer follow up. Future work should compare the pattern of prescription drug use between survivors and age-matched controls, and determine whether the difference in drug utilization might be attributable to cancer treatment-associated medical problems in survivors.

We adopted a more conservative definition of polypharmacy (≥5 concurrent chronic medications) in the primary analysis. In our sensitivity analysis, the prevalence estimate of polypharmacy was even higher at 22.5% when the alternative definition of polypharmacy (≥2 concurrent medications) was adopted for the pediatric subgroup. We speculate that conducting this sensitivity analysis is well justified, as one recent scoping review reported that more than 80% of the studies defined polypharmacy as ≥2 concurrent medications or therapeutic classes in pediatric patients with chronic illnesses (20). Notably, 15% of our pediatric survivors were found to have been prescribed two or more concurrent chronic medications. This estimate is comparable to the rate of polypharmacy previously reported in children with autism spectrum disorder, psychiatric conditions, and epilepsy (40–42). The definition of polypharmacy for the pediatric population should have a lower threshold number of medications than that for the adult population, as children are expected to have a lower disease burden than adults. However, the use of multiple therapeutic classes of medications is likely warranted in “complex chronic conditions” such as childhood cancer (43). Unfortunately, the term “polypharmacy” often carries a negative connotation in the literature. More efforts are needed to streamline medication utilization and polypharmacy research in pediatric patients with chronic diseases. The development of clinical guidelines should also take into consideration both the benefits (efficacy and synergistic effects) and harms (cost, adverse effects, pill burden, and non-adherence) of combining medications when defining pediatric polypharmacy in survivors of childhood cancer.

The long-term use of multiple medications is associated with the risk of adverse drug events and increase in healthcare costs in survivors of cancer (8, 13, 14). This is especially concerning in settings where the survivor may be prescribed other acute medications in addition to the regular chronic medications. For example, variability in the absorption and metabolism of oral levothyroxine can occur when it is administered with proton pump inhibitors, antacids, and certain fluoroquinolones (44). In addition to sex hormones and thyroid replacement therapies, late effects in HSCT recipients are typically treated with other chronic drugs such as steroid immunosuppressants, anxiolytics, antihypertensive drugs, and statins. One review examining post-HSCT sexual health highlighted that drug–drug interactions, pill burden, and combined adverse-effect profiles of medications may exacerbate sexual dysfunction in HSCT recipients (45). We propose that future work should evaluate health and humanistic outcomes associated with medication burden in high-risk groups of survivors. These studies will allow a better evaluation of potential drug-drug interactions and predictors of polypharmacy in this vulnerable population.

We inferred from our study findings that a closer monitoring of adverse health outcomes and drug interactions is warranted in survivors of CNS tumors and survivors who have undergone HSCT or cranial radiation. In terms of interventions, many studies on the cancer population have reported the benefits of having a pharmacist or interdisciplinary team to review medication use in patients with high medication burden (46–49). Such initiatives have successfully led to the reduction in potentially inappropriate or redundant medications, preventing drug interactions and promoting better medication-related behaviors in patients (50–52). Pharmacists as drug experts can perform routine medication reconciliation at every follow-up visit with the survivor and enquire about the use of over-the-counter drugs and CAM agents. This is especially relevant in the clinical setting of Hong Kong and China, where oncologists struggle with a heavy patient load. Furthermore, complementary/alternative medication (CAM) is becoming increasingly prevalent among children with cancer (53–56). Identifying clinical consequences of herb–drug interactions is an under-addressed problem in the field of cancer survivorship because few clinical trials have evaluated the concurrent use of CAM and Western medications in cancer survivors. There is a risk that CAM-related adverse events may not be detected or may be confounded with symptoms of late effects or adverse effects related to Western medications. Thus, pharmacists play an important role in counseling survivors on the appropriate use of medications and maintaining active communication between the oncologists and survivors regarding modifications to the medication list.

Despite having a relatively large, well-characterized sample with reliable sources of medication unitization data, the findings of this study should be considered in the context of several limitations. First, the CMS and CDARS databases of the Hospital Authority in Hong Kong vary in the quality of recorded health information (17). For example, late effects are dependent on the doses of radiation, but dosimetry data were not included in our study because of the inconsistency of available radiation records documented in our clinical databases. The calculated prevalence of chronic health conditions in our study cohort (10.9%) may not be a true reflection of the actual prevalence because survivors who presented with mild clinical presentation of late effects (e.g., Grade 1 conditions on the Common Terminology Criteria for Adverse Events Scale) may not have their conditions coded in the electronic health records. Further verification with the consultation notes is needed to validate disease coding in the CDARS. Second, based on geographical locations, there are seven clusters of public medical institutions in Hong Kong. The catchment area of the current study site included only the New Territories East Cluster. However, given the small cohort of healthcare professionals who specialize in pediatric oncology in Hong Kong, there is no reason to speculate that the treatment, healthcare, and drug utilization outcomes in our study cohort were any different from those in other clusters. Third, although we adjusted for demographic variables, medication use may not be directly attributable to the cancer treatment exposures, as there may be other confounding factors that were not captured in this study. For example, the relatively high rate of antihistamine use may be due to pre-existing allergic conditions that are not related to the cancer or the treatment exposures. Lastly, our estimation of medication burden did not include medication prescribed by private institutions, over-the-counter medications, and oral forms of CAM. Therefore, the true prevalence of polypharmacy may be even higher than the rate detected in this study.

## Conclusion

Our findings suggest that although polypharmacy was observed in a minority of the included survivors in this study, chronic medication use was common occurrence and has the potential to contribute to future medical burden. Consistent with the literature, we found that treatment-related chronic morbidities develop gradually in survivors as they age and may compound the occurrence of other age-related chronic health conditions. Our study identified a higher medication burden was more prevalent in survivors of CNS tumors and survivors who had undergone cranial radiation or HSCT. One potential clinical implication is that such individuals should be monitored more closely for drug–drug interactions and adverse health outcomes that may result from multiple chronic medications, particularly during hospitalization in an acute care setting. Given that this is a single-centered study with a modest sample size, our findings should be validated in larger cohorts of childhood cancer survivors. Future studies should also focus on identifying factors affecting prescribing behaviors and drug utilization patterns in this specific population. It is anticipated that such real-word data may help us understand healthcare resource utilization and the potential impact of newer treatments in survivors of childhood cancer.

## Data Availability Statement

The raw data supporting the conclusions of this article will be made available by the authors, without undue reservation.

## Ethics Statement

The studies involving human participants were reviewed and approved by The Joint Chinese University of Hong Kong-New Territories East Cluster Clinical Research Ethics Committee. Written informed consent from the participants’ legal guardian/next of kin was not required to participate in this study in accordance with the national legislation and the institutional requirements.

## Author Contributions

CE, YMC, HL, and YTC were responsible for the conceptualization and data analysis of the work. JW, AC, FP, and CL contributed in the study design and methodology. All authors contributed to the article and approved the submitted version.

## Funding

The research was supported by grant number 03170047 from the Food and Health Bureau Hong Kong (Health and Medical Research Fund - Research Fellowship).

## Conflict of Interest

The authors declare that the research was conducted in the absence of any commercial or financial relationships that could be construed as a potential conflict of interest.
