# Supplementary material for: Use of Chronic Prescription Medications and Prevalence of Polypharmacy in Survivors of Childhood Cancer
Source: Front Oncol. 2021 Apr 1;11:642544. doi: 10.3389/fonc.2021.642544 (PMC8047635; doi:10.3389/fonc.2021.642544)
Supplement: Supplementary file 1 [file DataSheet_1.docx]

Supplement 1: Excluded Medications without Active Ingredients

Aqueous Cream

Emulsifying Ointment

Liquid Paraffin

Paraffin Soft White

Hypromellose Eye Drops

Duratears Eye Ointment

Naturale Tears Free

Naturale Tears II

Naturale Tears Forte

Genteal Eye Gel

Carboxymethylcellulose Eye Drops

Systane Ultra Eye Drops

Normal saline

Chlorhexidine gluconate disinfectant solution

Olive oil

KY jelly

Supplement 2: ATC Code of the Top 10 Most Prevalent Therapeutic Classes

Therapeutic classes and specific medications prescribed to more than 5% of the cohort prescribed with chronic medications

| **ATC Code** | **Therapeutic Class** | **Drugs** |
| --- | --- | --- |
| R06 | Antihistamines for systemic use | Chlorpheniramine, Cetirizine, Dexchlorpheniramine, Loratadine |
| G03 | Sex hormones and modulators of the genital system | Testosterone, Estrogens Conjugated, Estradiol + Norethisterone Combined Pill, Medroxyprogesterone, Ethinylestradiol + Levonergestrel Combined Pill |
| H03 | Thyroid therapy | Thyroxine, Carbimazole |
| J01 | Antibacterials for systemic use | Cotrimoxazole, Doxycycline, Azithromycin, Phenoxymethylpenicillin |
| R03 | Drugs for obstructive airway diseases | Salbutamol Inhaler, Beclomethasone Inhaler, Budesonide + Formoterol Turbuhaler, Montelukast, |
| A02 | Drugs for acid related disorders | Famotidine, Esomeprazole, Aluminium Hydroxide + Magnesium Hydroxide + Simethicone Tablet |
| H01 | Pituitary and hypothalamic hormones and analogs | Desmopressin, Somatropin |

Supplement 3: Polypharmacy Stratified by Clinical and Treatment Variables

|  | **No Chronic Medications** | | **< 5 Chronic Medications** | | **Polypharmacy*** | |
| --- | --- | --- | --- | --- | --- | --- |
|  | n | % | n | % | n | % |
| Overall cohort (n=625) | 406 | 65.0 | 186 | 29.7 | 33 | 5.3 |
| **Demographic factors** |  |  |  |  |  |  |
| **Age at study** |  |  |  |  |  |  |
| ≥ 3 to ≤ 6 (n=22) | 12 | 54.5 | 9 | 40.9 | 1 | 4.5 |
| > 6 to ≤ 12 (n=134) | 88 | 65.6 | 38 | 28.3 | 8 | 5.9 |
| > 12 to ≤ 18 (n=146) | 89 | 60.9 | 50 | 34.2 | 7 | 4.7 |
| > 18 to ≤ 30 (n=306) | 209 | 68.3 | 82 | 26.8 | 15 | 4.9 |
| > 30 (n=17) | 8 | 47.0 | 7 | 41.1 | 2 | 11.7 |
| **Sex** |  |  |  |  |  |  |
| Male (n=358) | 234 | 65.3 | 109 | 30.5 | 15 | 4.2 |
| Female (n=267) | 172 | 64.4 | 77 | 28.8 | 18 | 6.7 |
| **Clinical factors** |  |  |  |  |  |  |
| **Age at diagnosis** |  |  |  |  |  |  |
| Pediatric (0 to < 15 years) (n=552) | 361 | 65.3 | 162 | 29.3 | 29 | 5.4 |
| Adolescent (≥ 15 to ≤ 18 years) (n=73) | 45 | 61.6 | 24 | 32.9 | 4 | 5.5 |
| **Time since diagnosis** |  |  |  |  |  |  |
| ≤ 5 years post-diagnosis (n=142) | 69 | 48.6 | 63 | 44.4 | 10 | 7.0 |
| > 5 years post-diagnosis (n=483) | 337 | 69.8 | 123 | 25.4 | 23 | 4.8 |
| **Time since EOT** |  |  |  |  |  |  |
| ≤ 2 (n=55) | 20 | 36.3 | 30 | 54.5 | 5 | 9.2 |
| ≤ 2 to 5 (n=115) | 68 | 59.1 | 41 | 35.7 | 6 | 5.2 |
| > 5 to ≤ 10 (n=175) | 127 | 72.5 | 38 | 21.7 | 10 | 5.7 |
| > 10 to ≤ 15 (n=159) | 109 | 68.5 | 43 | 27.0 | 7 | 4.4 |
| > 15 (n=121) | 82 | 67.7 | 34 | 28.1 | 5 | 4.1 |
| **Primary diagnosis** |  |  |  |  |  |  |
| Hematological (n=345) | 226 | 65.5 | 104 | 30.1 | 15 | 4.3 |
| CNS (n=66) | 26 | 39.3 | 31 | 46.9 | 9 | 13.6 |
| Non-CNS solid tumor (n=214) | 154 | 71.9 | 51 | 23.8 | 9 | 4.2 |
| **Chronic health conditions (any)** |  |  |  |  |  |  |
| Yes (n=68) | 20 | 29.4 | 34 | 50.0 | 14 | 20.6 |
| No (n=557) | 386 | 69.3 | 152 | 27.2 | 19 | 3.4 |
| **Treatment factors** |  |  |  |  |  |  |
| **Chemotherapy** |  |  |  |  |  |  |
| Yes (n=557) | 357 | 64.1 | 172 | 30.9 | 28 | 5.0 |
| No (n=68) | 49 | 72.0 | 14 | 20.6 | 5 | 7.3 |
| **Radiation** |  |  |  |  |  |  |
| Cranial radiation (n=73) | 34 | 46.5 | 27 | 37.0 | 12 | 16.4 |
| Other body sites (n=75) | 45 | 60.0 | 26 | 34.6 | 4 | 5.3 |
| None (n=477) | 327 | 68.5 | 133 | 27.8 | 17 | 3.5 |
| **Surgery** |  |  |  |  |  |  |
| Yes (n=265) | 169 | 63.8 | 81 | 30.6 | 15 | 5.6 |
| No (n=360) | 237 | 65.8 | 105 | 29.2 | 18 | 5.0 |
| **Bone Marrow Transplant** |  |  |  |  |  |  |
| Yes (n=75) | 33 | 44.0 | 32 | 42.7 | 10 | 13.3 |
| No (n=550) | 373 | 67.8 | 164 | 28.0 | 23 | 4.18 |

CNS: central nervous system; EOT: end of treatment; ref: reference group

* Polypharmacy defined as “≥5 concurrent medications”

Supplement 4: Cox Regression Model for Polypharmacy

Model: *P*<.0001


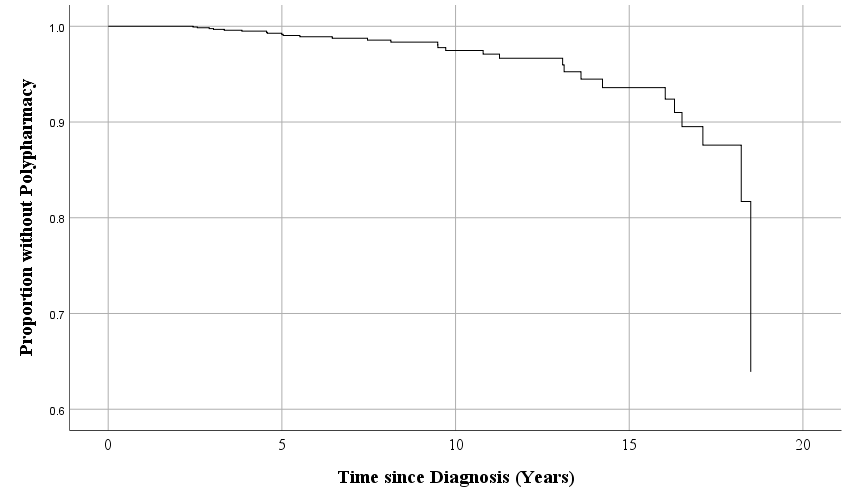


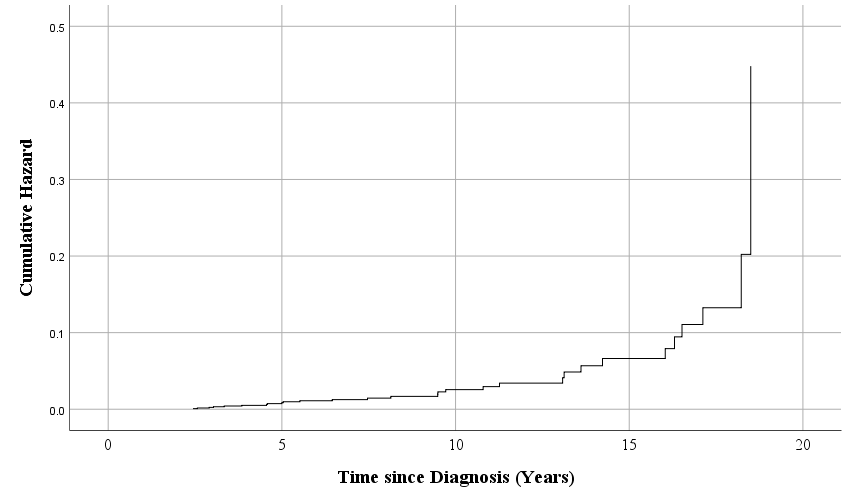


|  | **Hazard Ratio** | **95% CI** | | ***P*** |
| --- | --- | --- | --- | --- |
| **Demographic factors** |  |  |  |  |
| **Sex** |  |  |  |  |
| Male | 0.67 | 0.33 | 1.36 | 0.27 |
| Female | *Ref* |  |  |  |
| **Clinical factors^** |  |  |  |  |
| **Primary diagnosis** |  |  |  |  |
| CNS | 1.79 | 1.25 | 5.07 | **0.042** |
| Non-CNS solid tumor | *Ref* |  |  |  |
| **Chronic health conditions (any)** |  |  |  |  |
| Yes | 3.39 | 1.57 | 7.34 | **0.002** |
| No | *Ref* |  |  |  |
| **Treatment factors^** |  |  |  |  |
| **Radiation** |  |  |  |  |
| Cranial radiation | 3.34 | 1.22 | 9.13 | **0.018** |
| None | *Ref* |  |  |  |
| **Bone Marrow Transplant** |  |  |  |  |
| Yes | 2.58 | 1.19 | 5.62 | **0.016** |
| No | *Ref* |  |  |  |

Supplement 5: Factors Associated with Polypharmacy* (Sensitivity Analysis)

|  | **Risk Ratio** | **95% CI** | | ***P*** |
| --- | --- | --- | --- | --- |
| **Demographic factors** |  |  |  |  |
| **Sex** |  |  |  |  |
| Male | 0.81 | 0.53 | 1.26 | 0.35 |
| Female | *Ref* |  |  |  |
| **Current age at study** | 1.05 | 1.02 | 1.09 | **0.0012** |
|  |  |  |  |  |
| **Clinical factors^** |  |  |  |  |
| **Age at diagnosis** | 1.01 | 0.95 | 1.07 | 0.61 |
| **Years since EOT** | 0.97 | 0.91 | 1.03 | 0.36 |
| **Primary diagnosis** |  |  |  |  |
| Hematological | 1.22 | 0.74 | 2.00 | 0.43 |
| CNS | 2.37 | 1.19 | 4.72 | **0.014** |
| Non-CNS solid tumor | *Ref* |  |  |  |
| **Chronic health conditions (any)** |  |  |  |  |
| Yes | 4.930 | 2.78 | 8.73 | **<.0001** |
| No | *Ref* |  |  |  |
| **Treatment factors^** |  |  |  |  |
| **Chemotherapy** |  |  |  |  |
| Yes | 0.56 | 0.25 | 1.24 | 0.15 |
| No | *Ref* |  |  |  |
| **Radiation** |  |  |  |  |
| Cranial radiation | 3.33 | 1.80 | 6.17 | **0.0001** |
| Body only (Chest, abdomen, pelvis) | 1.64 | 0.85 | 3.15 | 0.13 |
| None | *Ref* |  |  |  |
| **Surgery** |  |  |  |  |
| Yes | 1.01 | 0.65 | 1.57 | 0.95 |
| No | *Ref* |  |  |  |
| **Bone Marrow Transplant** |  |  |  |  |
| Yes | 3.00 | 1.69 | 5.31 | **0.0002** |
| No | *Ref* |  |  |  |

CNS: central nervous system; EOT: end of treatment; ref: reference group

*Polypharmacy defined as “≥5 concurrent medications” in adult survivors (>18 years of age), and “≥2 concurrent medications” in pediatric survivors (≤18 years of age)

^Models are adjusted for demographic factors: sex and age at study.

Supplement 6: Association of Age at Diagnosis and Time since Diagnosis with Polypharmacy* (Sensitivity Analysis)

|  | **Risk Ratio**^ | **95% CI** | | ***P*** |
| --- | --- | --- | --- | --- |
| **Age at diagnosis** |  |  |  |  |
| Adolescent (≥ 15 to ≤ 18 years) | 1.11 | 0.34 | 3.56 | 0.86 |
| Pediatric (0 to < 15 years) | *Ref* |  |  |  |
| **Time since diagnosis** |  |  |  |  |
| ≤ 5 years post-diagnosis | 2.08 | 0.84 | 5.16 | 0.11 |
| > 5 years post-diagnosis | *Ref* |  |  |  |

Ref: reference group

* Polypharmacy defined as “≥5 concurrent medications”

^Models are adjusted for demographic factors: sex and age at study
